# Intraoperative measurements of reverse total shoulder arthroplasty contact forces

**DOI:** 10.1186/s40634-020-00311-0

**Published:** 2020-12-08

**Authors:** Kevin W. Farmer, Masaru Higa, Scott A. Banks, Chih-Chiang Chang, Aimee M. Struk, Thomas W. Wright

**Affiliations:** 1 Department of Orthopaedics and Rehabilitation, University of Florida, 3450 Hull Road, 3rd Floor, Gainesville, FL 32608 USA; 2 University of Hyogo, Himeji, Japan; 3 Department of Mechanical & Aerospace Engineering, University of Florida, MAE-A 318, Gainesville, FL 32611-6250 USA; 4 Orthopaedics and Sports Medicine Institute, University of Florida, 3450 Hull Road, Gainesville, FL 32611 USA

**Keywords:** Intraoperative glenohumeral contact forces, Reverse total shoulder arthroplasty, Intraoperative tensioning, Abduction, External rotation, Scaption

## Abstract

**Purpose:**

Instability and fractures may result from tensioning errors during reverse total shoulder arthroplasty (RTSA). To help understand tension, we measured intraoperative glenohumeral contact forces (GHCF) during RTSA.

**Methods:**

Twenty-six patients underwent RTSA, and a strain gauge was attached to a baseplate, along with a trial glenosphere. GHCF were measured in passive neutral, flexion, abduction, scaption, and external rotation (ER). Five patients were excluded due to wire issues. The average age was 70 (range, 54–84), the average height was 169.5 cm (range, 154.9–182.9), and the average weight was 82.7 kg (range, 45.4–129.3). There were 11 females and 10 males, and thirteen 42 mm and 8 38 mm glenospheres.

**Results:**

The mean GHCF values were 135 N at neutral, 123 N at ER, 165 N in flexion, 110 N in scaption, and 205 N in abduction. The mean force at terminal abduction is significantly greater than at terminal ER and scaption (*p* < 0.05).

**Conclusions:**

These findings could help reduce inappropriate tensioning.

## Background

Reverse total shoulder arthroplasty (RTSA) has become a widely used treatment for a variety of conditions, including cuff-tear arthropathy [5], pseudoparesis due to massive rotator cuff tear [29], fracture [7], rheumatoid arthritis [35], revision of failed total shoulder arthroplasty [33], and osteoarthritis with glenoid wear [17]. Despite the increasingly widespread use and excellent short-term outcomes, there are numerous studies detailing the complications associated with RTSA. Recurrent prosthetic instability accounts for a large percentage of these complications in some studies [9, 11, 14]. Dislocation rates ranging from 2.4% to 31% have been reported, and early dislocation rates (< 3 months postoperative) have been reported at 2.9% [7–10, 18, 34]. The etiology of recurrent RTSA instability may be multifactorial, but one factor often cited is inadequate soft-tissue tension [9, 16, 20, 31]. In his groundbreaking article, Grammont coined the term “global decoaptation,” and described the situation as recurrent instability due to lack of sufficient deltoid tension [16, 19]. Intraoperative assessment of soft-tissue tension remains subjective to this day, with no reproducible method of assessment.

Although insufficient soft-tissue tensioning remains problematic, excessive soft-tissue tension can lead to complications as well. Complications of excessive soft-tissue tensioning include acromial fracture [26], brachial plexus injury [24], and excessive shear force on the glenoid/baseplate interface [1]. Postoperative acromial fractures have been reported following RTSA from 1% to 7% of patients [4, 12, 13, 15, 21–23, 26, 32, 34]. These fractures are thought to be secondary to increased stress and tension on the deltoid [21, 23, 26]. Acromial fractures can lead to a painful shoulder and decreased functional outcomes, but, to date, the role of soft-tissue tension in their development is not well understood.

Biomechanical studies using cadavers [1] and motion capture [28] have been developed that model glenohumeral contact forces (GHCF) following RTSA. To our knowledge, no studies have directly measured intraoperative GHCF. There have been numerous studies looking at hip and knee contact forces using instrumented implants [25]. Bergman et al. measured GHCF following implantation of an instrumented hemiarthroplasty 7 months postoperatively [2]. No similar studies have been performed looking at RTSA implants.

The purpose of this study was to measure the intraoperative GHCF during passive range of motion during implantation of a RTSA. This data could help provide valuable information for proper intraoperative soft-tissue tensioning. Having the ability to assess intraoperative soft-tissue tension better could lead to better outcomes and fewer complications following RTSA.

## Methods

### Data collection

After obtaining institutional review board approval, 26 patients with a planned primary RTSA were enrolled in this study. Inclusion criteria were patients undergoing primary RTSA. Exclusion criteria were revision cases, acute fractures, and severe bony deformity. Five of the 26 patients’ data were not available due to technical problems (mostly broken wires), leaving 21 shoulders in 21 patients in our data series.

### Measurements

**Fig. 1:**
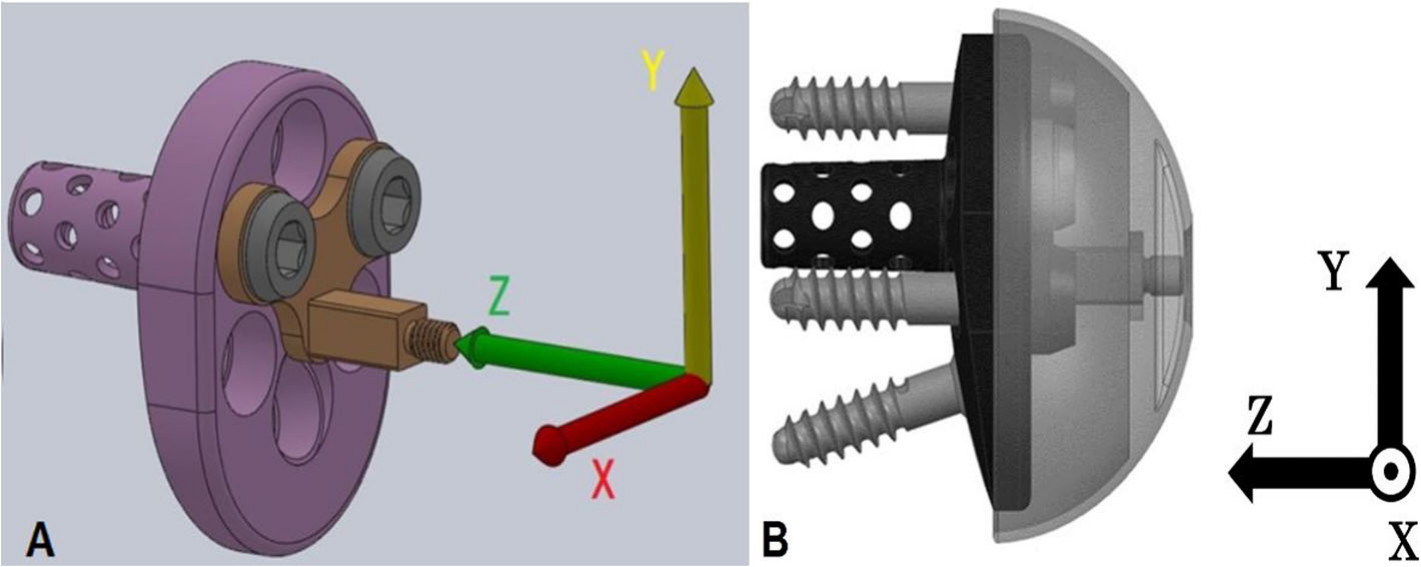
**a**-**b**. An instrumented trial implant is shown with a measure coordinate system. The X-axis directs anterior, Y superior, and Z medial **(a)**. The glenosphere is transparent to make the inside visible **(b)**

Measurements were comprised of the collection of intraoperative joint forces and motions. For joint force measurements, an instrumented trial implant of the RTSA system (EQUINOXE, Exactech, Inc., Gainesville, FL) was used, which has been validated in a cadaveric model [27]. The trial instrumented implant was designed to attach to the implanted baseplate already fixed to the glenoid. The specially designed trial glenosphere was then fixed to the instrumented implant (Fig. 1a-b). The outer dimensions of the instrumented trial implant are identical to those of the prosthesis that is used clinically [30]. Four uniaxial foil strain gauges (QFLG-02-11-3LJB, Tokyo Sokki Kenkyujo Co., Ltd., Japan) were placed inside the instrumented trial implant. Wires from the strain gauges were run outside the operative field and connected to a 24-bit analog input module (NI9237, National Instruments, Co., Ltd.). The instrumented trials were packaged and sterilized as is required for standard clinical use. The strain gauges and coating materials were single use and disposable.

**Fig. 2:**
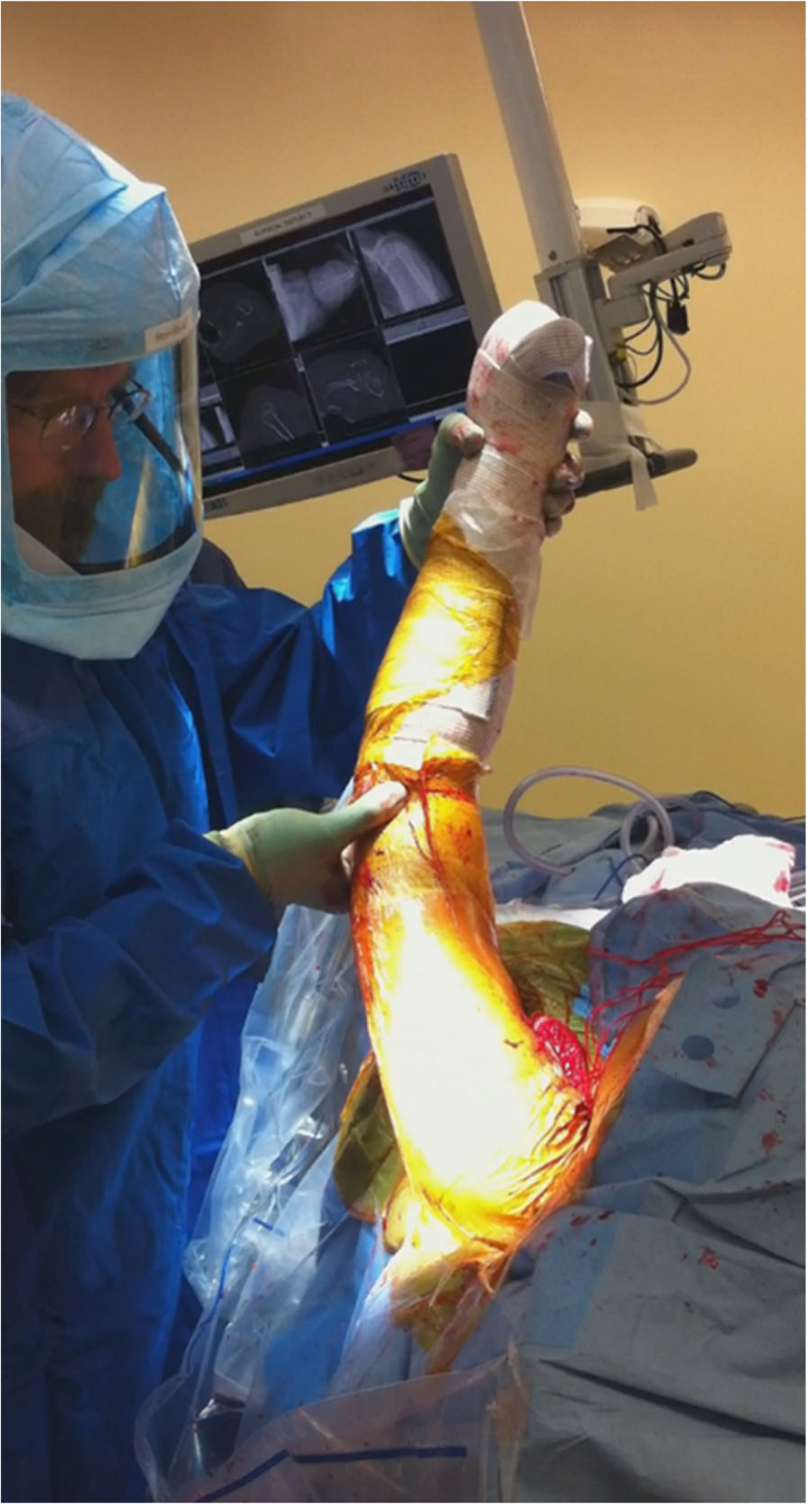
The patient’s shoulder was moved five times in ER, flexion, scaption, and abduction

After reduction of the RTSA, forces were measured in 0 degrees abduction/flexion/external rotation (ER), defined as “neutral.” Next, force measurements were taken during 5 repetitions of full ER from the neutral position. GHCF measurements were then subsequently measured during 5 repetitions of full flexion, full scaption, and full abduction (Fig. 2).

### Postoperative calibration and data processing

Washing and sterilization of the sensor may affect the measurement performance, so post-use calibrations were performed after each clinical use. The external force vector (Fx, Fy, Fz) can be expressed in terms of the strain gauge outputs as follows:

1 $$ \left[\begin{array}{l}{F}_x\\ {}{F}_y\\ {}{F}_z\end{array}\right]=T\left[\begin{array}{l}{S}_1\\ {}{S}_2\\ {}{S}_3\\ {}{S}_4\end{array}\right] $$

where T is a calibration matrix, and Si (i = 1 to 4) corresponds to the outputs of the four strain gauges. The calibration matrices were calculated according to well-established methods [3] for every sensor after use in surgery. The calibration procedures conformed to ASTM E-4 standards (ASTM, 2008).

Force values at neutral position, terminal ER, terminal flexion, terminal scaption, and terminal abduction were analyzed by use of one-way ANOVA to determine whether there were any significant differences in joint reaction force and arm position. A post hoc analysis was also applied. Tukey-Kramer methods were used when multiple comparisons were made.

### Operative technique

All cases were done through a deltopectoral approach. If the subscapularis was intact, it was tenotomized during the approach. It was not repaired prior to GHCF testing. All patients received regional and general anesthesia, and confirmation of zero muscular twitches was confirmed by anesthesia prior to trialing as per our typical trialing protocol. A humeral head cut was performed at or just below the anatomic neck in all cases. The final humeral stem (Exactech, Inc., Gainesville FL) was placed after appropriate reaming and broaching. The glenoid baseplate was placed after glenoid preparation in neutral-to-slightly inferior tilt. Care was taken to ensure the inferior edge of the baseplate was at or just below the inferior lip of the glenoid. The instrumented implant was then placed, and testing began. Typically, females received a 38 mm glenosphere and males received a 42 mm glenosphere, unless preoperative or intraoperative factors led to a change in implant size. Trial trays were chosen based on the surgeon’s subjective feeling of appropriate stability.

## Results

### Patient/implant characteristics

Twenty-one shoulders in 21 patients completed the study. The average patient age was 70.1 years (range, 54–84). Their average height was 169.5 cm (range, 154.9–182.9 cm), and average weight was 82.7 kg (range, 45.4–129.3 kg). There were 11 females and 10 males. There were thirteen 42 mm glenospheres and eight 38 mm glenospheres used during testing. Sixteen RTSAs were performed for cuff-tear arthopathy, 1 for irreparable rotator cuff tears, 1 for proximal humeral malunion, 1 for glenohumeral arthritis, and 1 for rheumatoid arthritis.

### Force/motion data

**Fig. 3:**
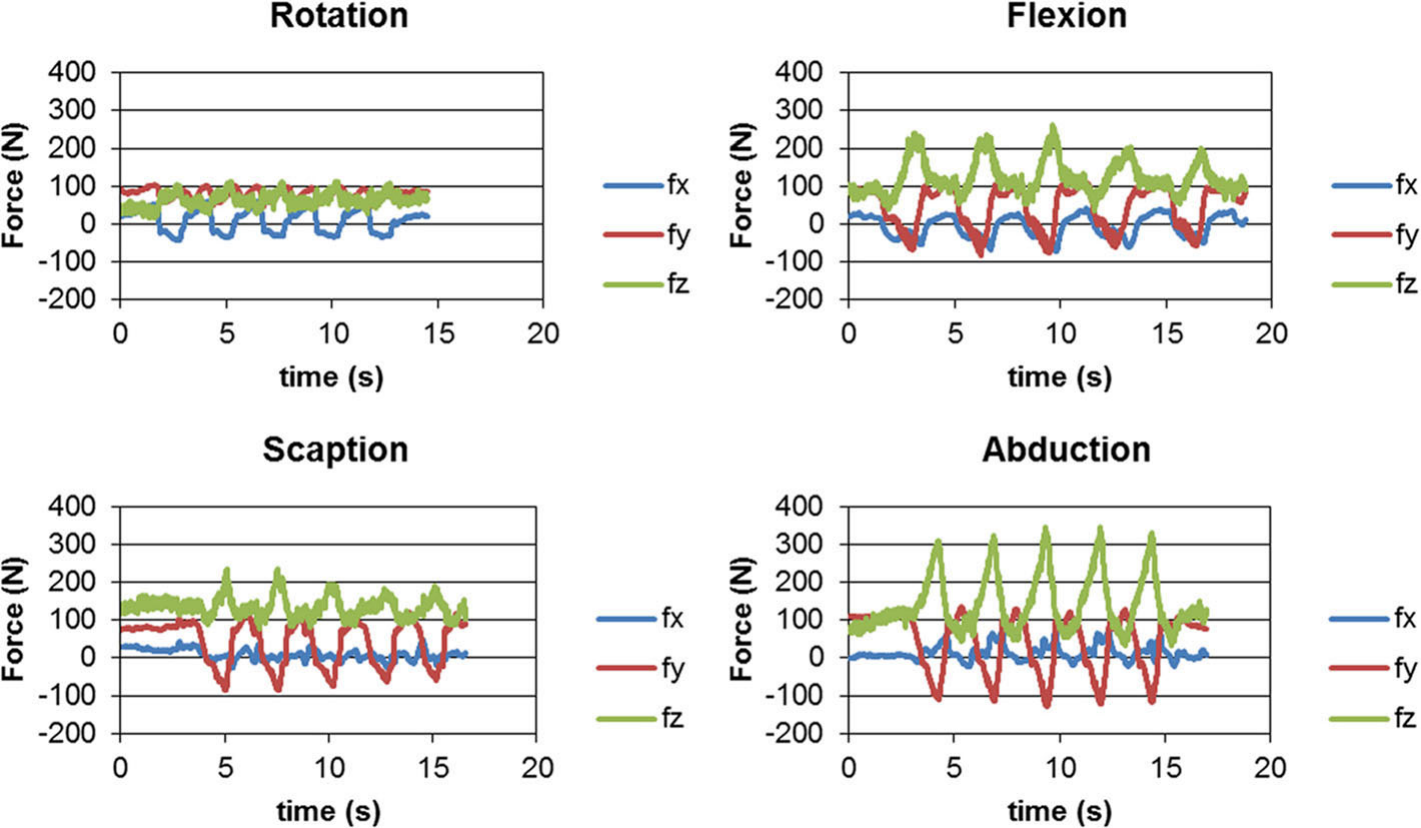
Force curves are shown at the four identified movements. X, Y, Z directions correspond to the coordinate system in Fig. 2

**Fig. 4:**
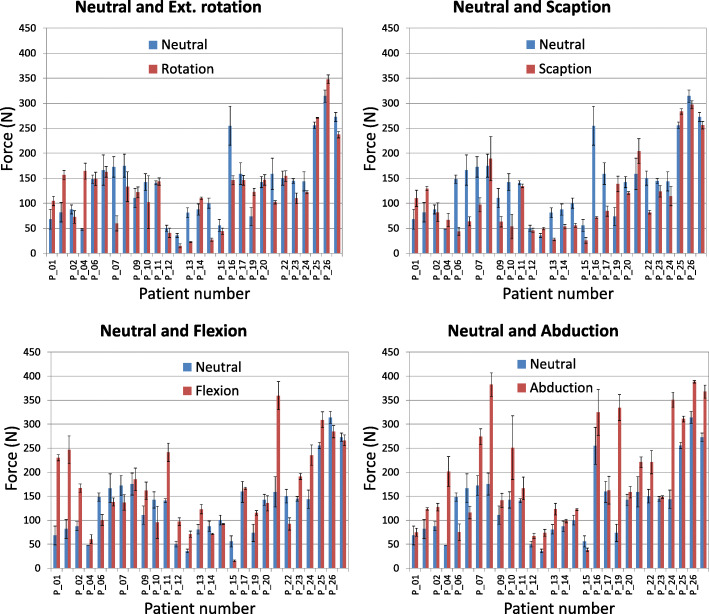
Resultant forces are shown at both the neutral position (blue) and after each movement (orange) for all patients. Error bars are SD of the five measurements

**Fig. 5:**
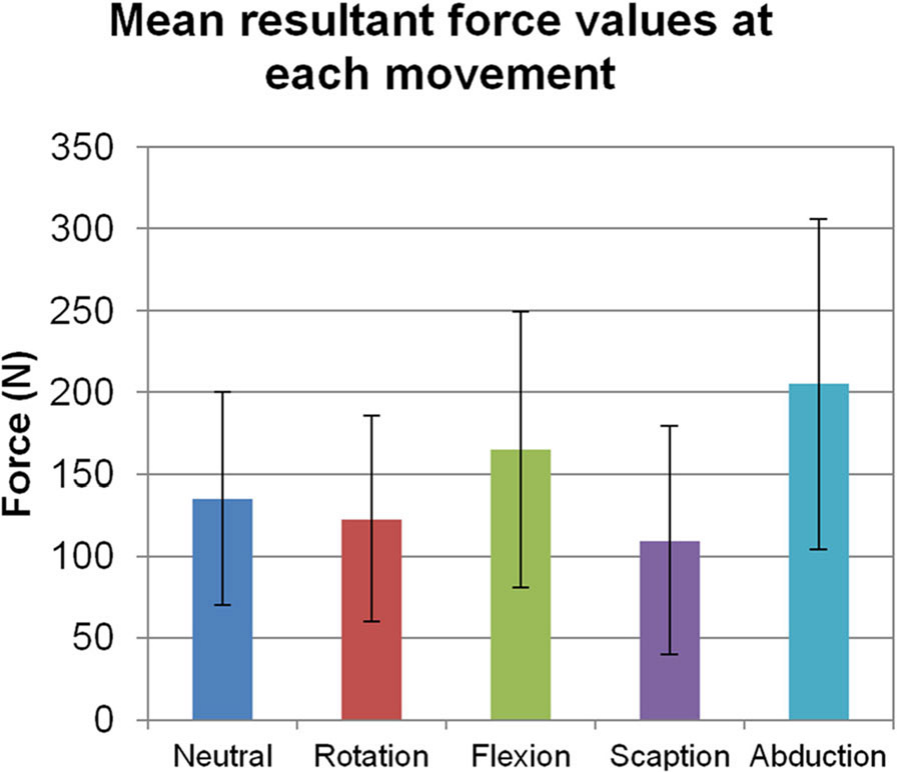
Force values of all patients are averaged at each identified motion. Error bars are SD of inter-subjects deviations. Mean force for scaption and ER is significantly less than abduction (*p* < 0.05)

One of the representative force data sets of a patient for the identified motions is noted in Fig. 3. Force directions were defined by the force exerted by the humeral tray on the glenosphere. Positive values indicate that the humeral tray pushes the glenosphere towards positive X direction (nearly anterior on the left shoulder), towards positive Y direction (nearly superior), and towards positive Z direction (nearly medial). Conversely, negative values mean opposite directions. Mean force components (Fx, Fy, Fz) [N] were (− 6, 76, 80) N in neutral position, (6, 65, 64) N in external rotation, (− 48, 14, 139) N in flexion, (− 2, − 18, 81) N in scaption, and (53, − 24, 155) N in abduction, respectively. The resultant force values at terminal ER, terminal flexion, terminal scaption, and terminal abduction were read and averaged, as well as force values at the neutral position for every patient (Fig. 4). Mean (SD) resultant force values were 135 N (65) of neutral, 123 N (63) of ER, 165 N (85) of flexion, 110 N (70) of scaption, and 205 N (101) of abduction (Fig. 5). Mean SDs within subjects for 5 repeated cycles of an activity were 11 N for external rotation, 14 N for flexion, 12 N for scaption, and 18 N for abduction, respectively. The mean force values are significantly affected by joint position (*p* = 0.002). The mean force at terminal abduction is significantly greater than the mean forces at terminal ER and terminal scaption (*p* < 0.05).

## Discussion

The most important finding of this study was that soft-tissue glenohumeral contact forces were at their lowest during scaption and ER, and at their greatest during abduction. RTSA has become a popular and effective option for treating a variety of pathologies. Despite its success and improving outcomes, complications from deficient soft-tissue tensioning (instability) and excessive tensioning (acromial fracture, neurologic injury) still occur. Currently, surgeons have very little objective information when deciding on the appropriate soft-tissue tensioning intraoperatively. Subjective guidelines such as assessing conjoined tendon or deltoid tension have been described [5]. Some surgeons use the ease of dislocation at various joint angles as their method of assessing stability. To our knowledge, this study is the first to quantify forces intraoperatively, and the first to assess GHCF during passive range of motion of the shoulder.

In our study, joint position was significantly associated with GHCF. Compared to the neutral position, GHCF were decreased with scaption and ER, and increased with abduction. GHCF during abduction were significantly greater than scaption or ER. Surgeons often assess tension at neutral by checking longitudinal traction (“shuck”), and at varying degrees of ER/extension, which also assesses the risk of posterior impingement. Based on the data from this study, it may be more reasonable to assess stability in varying degrees of scaption and ER. Shuck and ease of dislocation may increase with either ER or scaption, compared to neutral. In addition, the intraoperative data demonstrated that GHCF are greatest in abduction. Surgeons should keep this in mind and assess the soft-tissue tension in full abduction when trialing, in addition to neutral where it is commonly assessed.

To our knowledge, there are no comparable studies of RTSA intraoperative contact forces. Ackland et al. looked at GHCF in a RTSA cadaver model. They found a joint force magnitude of 84.5% body weight at 75° abduction [1]. Studies looking at native GHCF in active abduction demonstrate GHCF between 420 and 600 N [2, 6, 30]. Although none of these studies are directly comparable to our data, they do demonstrate that intraoperative RTSA GHCF are greatly reduced compared to previously published data in loaded shoulders. This may indicate that overtensioning in the operating room could be further detrimental as GHCF will likely be greater with increased muscle tone and active range of motion.

Instrumented implants have been previously used to assess contact forces following arthroplasty. Multiple studies have measured hip joint contact forces using an implantable instrumented hip prosthesis [25], some studies for up to a decade. Bergmann et al. looked at an instrumented hemiarthroplasty and found that, in 75° abduction, the force resultant is 85% of body weight [2]. Custom instrumented tibial inserts have been used for measuring joint forces following total knee arthroplasty [25]. These studies provide support to this method of assessing joint contact forces. The potential clinical relevance of this study could be to improve outcomes and decrease complications by guiding surgeons in how to assess soft-tissue tensioning in the appropriate arm positions.

The strengths of this study are that glenohumeral contact forces are measured using an intraoperative tensionometer. The are positions are carefully recorded, and the arm position are correlated to contact forces. This novel approach provides real-time contact-force data in arm positions, and provides useful information as to the best time to assess stability.

There are several limitations to our study. First, all data were collected intraoperatively. In this setting, most patients were at or near muscle paralysis. Certainly, the GHCF taken intraoperatively are greatly reduced compared to forces postoperatively. An implantable device that could measure forces during daily activity would provide valuable information. In addition, we did not repair any subscapularis tendons prior to testing. It is likely that subscapularis repair may alter the findings of GHCF. All implants were Exactech Equinoxe RTSA (Exactech, Inc., Gainesville, FL). This prosthesis has a medial center of rotation but a lateralized humerus. The GHCF data from this implant may not be applicable to other implants with medial humeral designs on the market. In our institution, females most commonly receive a 38-mm glenosphere and males typically a 42-mm glenosphere. Changes in glenosphere size can alter GHCF, but we elected to remain consistent with our typical surgical technique to not alter the soft-tissue tension we typically assess. Also, the instrumented implant assesses the force acting on the glenosphere and the baseplate. It is assumed that this force is predominantly from glenosphere-polyethylene loading, but muscles and capsule wrapping around the glenosphere could contribute to the measured forces.

In addition, we did not break down forces based on diagnosis or rotator cuff integrity. It is possible that some of the variation in GHCF may be due to differences based on the underlying diagnosis. Future, larger studies may be helpful to break down the forces seen in different diagnoses.

Lastly, the appropriate implants at the time of trialing were made based on the surgeons’ subjective assessment of stability. There is likely a range of GHCF that occur in a “stable” prosthesis, which could explain some of the variation seen in GHCF in different patients. We are hopeful that more objective criteria of stability will be developed in the future, and that this type of study will be beneficial in developing them.

## Conclusions

Despite these limitations, we believe our data provide proof of principle that intraoperative joint force measurements can be performed during routine RTSA procedures. A surgeon’s only opportunity to achieve optimal shoulder joint tensioning is during surgery, and objective intraoperative measures will facilitate that goal.

GHCF vary based on intraoperative joint position following RTSA. Forces are at their lowest in scaption or ER. Forces are at their greatest during abduction. Surgeons should use this knowledge when assessing stability and soft-tissue tension intraoperatively. This information will hopefully be useful in improving outcomes following RTSA. Under-tensioned RTSA can lead to dislocation and need to revision surgery, over-tensioned RTSA can lead to the debilitating complication of acromial stress fractures. Surgeons should use the findings of this study to help assess soft-tissue tension in the appropriate arm position. Further follow-up studies are on-going to see if the outcomes are improved by utilizing the knowledge gained by this study.

## Data Availability

The datasets used and/or analysed during the current study are available from the corresponding author on reasonable request.

## Abbreviations

ER: External rotation
GHCF: Glenohumeral contact forces
RTSA: Reverse total shoulder arthroplasty
